# Bilateral and Unilateral Fused Primary Teeth With Hypodontia in Permanent Dentition: A Case Series

**DOI:** 10.7759/cureus.92772

**Published:** 2025-09-20

**Authors:** Rahaf W Budayri, Majd Morad, Rawan Alhazmy, Afnan S Aljohani

**Affiliations:** 1 Dentistry, Faculty of Dental Medicine, Umm Al-Qura University, Makkah, SAU; 2 Pediatric Dentistry, Al Noor Specialist Hospital, Makkah, SAU; 3 Pediatric Dentistry, Security Forces Hospital, Mecca, SAU

**Keywords:** dental fusion, developmental anomely, double tooth, hypodontia, pediatric dentistry, primary teeth

## Abstract

Fused teeth represent an uncommon developmental anomaly in which adjacent tooth buds unite to form a single enlarged structure, often leading to spacing discrepancies, malocclusion, esthetic concerns, and a frequent absence of permanent successors. Although more prevalent in the primary dentition, bilateral involvement is exceedingly rare and poses additional diagnostic and treatment challenges. This report describes three pediatric cases of mandibular primary tooth fusion associated with hypodontia of the successor incisors, confirmed radiographically. These cases underscore the importance of early clinical and radiographic evaluation, multidisciplinary management, and long-term follow-up to minimize complications and support optimal dental development.

## Introduction

Developmental dental anomalies that occur during the morphodifferentiation stage can affect both the primary and permanent dentition. These anomalies include gemination, fusion, and concrescence [1]. Differentiating between gemination and fusion can be challenging. Fused teeth are defined as a developmental anomaly in which two adjacent tooth buds partially or completely unite to form a single enlarged tooth structure through the confluence of dentin or enamel. This may involve the crown, the root, or both. In contrast, gemination occurs when a single tooth bud attempts to divide into two, resulting in two partially separated crowns that share a common root and pulp system. Clinically, fusion may reduce the number of teeth in the arch, depending on the extent of union [2].

Gemination and fusion are among the more frequently observed developmental anomalies in the primary dentition, particularly in the anterior region. Because of their morphological overlap, the term double tooth is often used in the literature as a general descriptor when the distinction is uncertain [3]. Among these anomalies, fusion is more frequently observed in the primary dentition (2.5%) than in the permanent dentition (0.5%). Bilateral fusion in primary teeth is extremely rare (0.02%), and when it occurs, it most often involves the mandibular dentition, with no significant sex predilection reported [1-4].

A recent nationwide study in Saudi Arabia on 3,000 children aged six to 18 years reported a prevalence of tooth fusion of only 0.17%, confirming its rarity [5]. Clinically, fused teeth may result in abnormal spacing, arch asymmetry, esthetic concerns, and a higher risk of caries due to deep developmental grooves. Importantly, they are strongly associated with anomalies in the permanent successors, including agenesis and malformation [6,7]. Studies have reported a high prevalence of missing permanent mandibular lateral incisors in cases of fusion, in some reports reaching up to 75% [8]. Early clinical and radiographic evaluation is therefore essential for timely diagnosis, space management, and long-term planning.

Tsujino et al. classified fusion in the primary dentition into three types: the first type is fusion between maxillary central and lateral incisors, the second type is fusion between mandibular central and lateral incisors, and the third type is fusion between the mandibular lateral incisor and canine. Among these, fusion involving the mandibular lateral incisor and canine, though less common, has the most profound clinical impact. This type is frequently associated with agenesis or ectopic eruption of the corresponding permanent lateral incisor or canine, often resulting in midline asymmetry, space discrepancies, and malocclusion that necessitate early orthodontic intervention and, in some cases, surgical management [9].

Fusion may also be categorized based on the stage of calcification. Complete fusion occurs when the union begins before calcification, leading to a crown with combined enamel, dentin, cementum, and pulp. Incomplete fusion occurs later, where crowns may remain separate but the roots are joined, with fused or distinct pulp canals [10].

This paper reports three clinical cases of mandibular fusion between the primary lateral incisor and canine, representing the third type of fusion. All cases were associated with congenital absence of the permanent mandibular lateral incisors. While unilateral fusion is uncommon, bilateral involvement in the primary dentition is exceptionally rare, underscoring the clinical significance of this case series. This report aims to document these unusual cases and highlight their impact on the developing permanent dentition.

## Case presentation

Three pediatric patients were seen at the Pediatric Dental Clinics of Security Forces Hospital (SFH), Makkah, Saudi Arabia, presenting with fused primary teeth. In all cases, the fusion involved the mandibular primary lateral incisor and canine, with a consistent association of congenitally missing permanent lateral incisors. 

Case one

**Figure 1:**
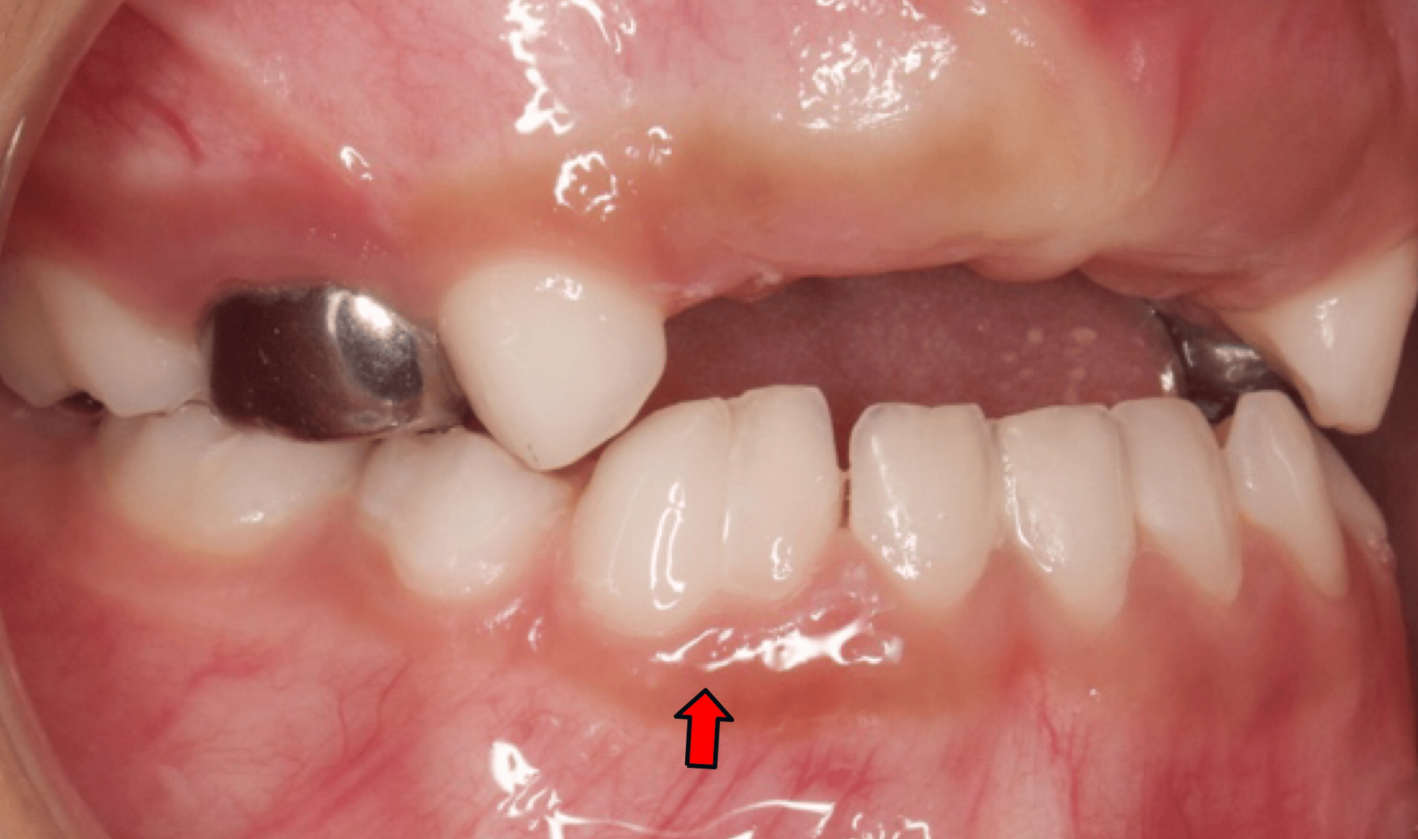
Intraoral view of case 1 The red arrow indicate unilateral fusion between mandibular primary lateral incisor and canine at the right side

A six-year-old girl was referred for dental treatment under general anesthesia due to her uncooperative behavior and extensive treatment needs. Her medical history was non-contributory, with no systemic conditions, family history of dental anomalies, parental consanguinity, or history of trauma. Clinical examination revealed poor oral hygiene, multiple carious teeth, and a unilateral fusion between the mandibular right primary lateral incisor and canine. The intraoral view clearly demonstrated the fused crowns, with the red arrow indicating the union between the two teeth (Figure 1).

**Figure 2:**
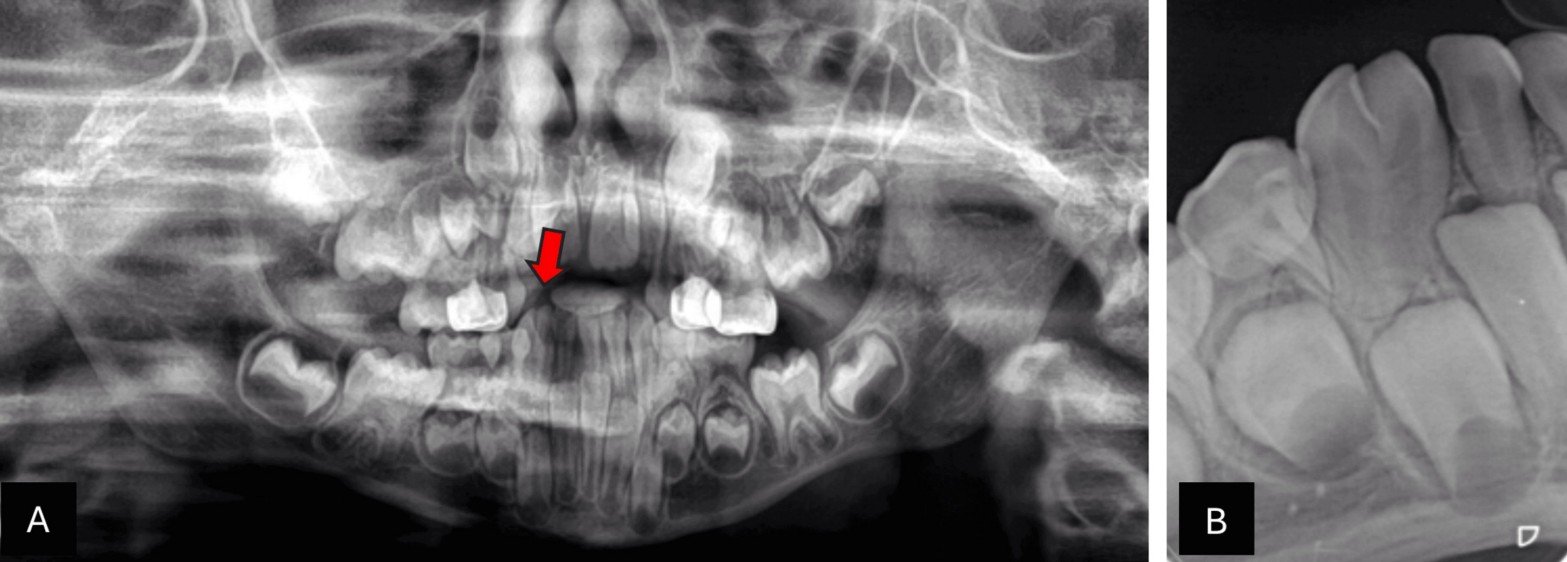
Radiographic examination of case 1 (A) Panoramic radiograph: the red arrow points to the unilateral fusion at the mandibular right side with congenitally missing successor lateral incisors. (B) Periapical radiograph: shows fusion between primary lateral incisor and canine with congenitally missing successor lateral incisors.

Radiographic evaluation confirmed the presence of two separate roots and demonstrated the congenital absence of the permanent mandibular right lateral incisor. The panoramic radiograph highlighted the unilateral fusion at the mandibular right side, with the red arrow pointing to the fused teeth (Figure 2A). The periapical radiograph further illustrated the fusion between the primary lateral incisor and canine, along with the absence of the permanent successor (Figure 2B).

Case twp

**Figure 3:**
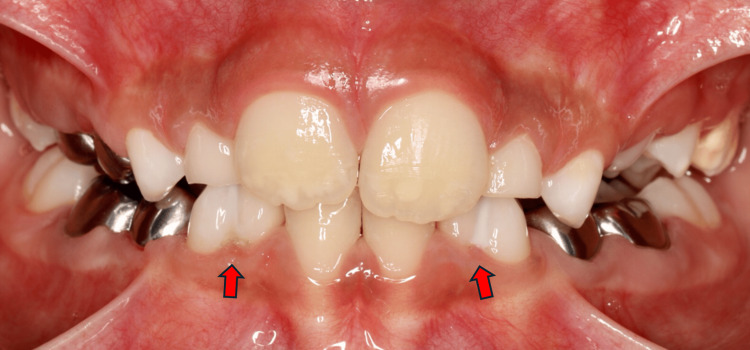
Intraoral view case 2 The red arrows highlight the bilateral fusion at mandibular arch between primary lateral incisors and canine on both sides.

A nine-year-old boy who presented with pain in his upper right primary molars. His medical and dental histories were unremarkable. Clinical examination revealed extremely poor oral hygiene, multiple carious teeth, and no previous dental treatment. Bilateral fusion was noted in the mandibular anterior region, specifically between the primary lateral incisors and canines. The intraoral photograph clearly demonstrates the bilateral fusion, with red arrows highlighting the union of the fused teeth on both sides of the mandibular arch (Figure 3).

**Figure 4:**
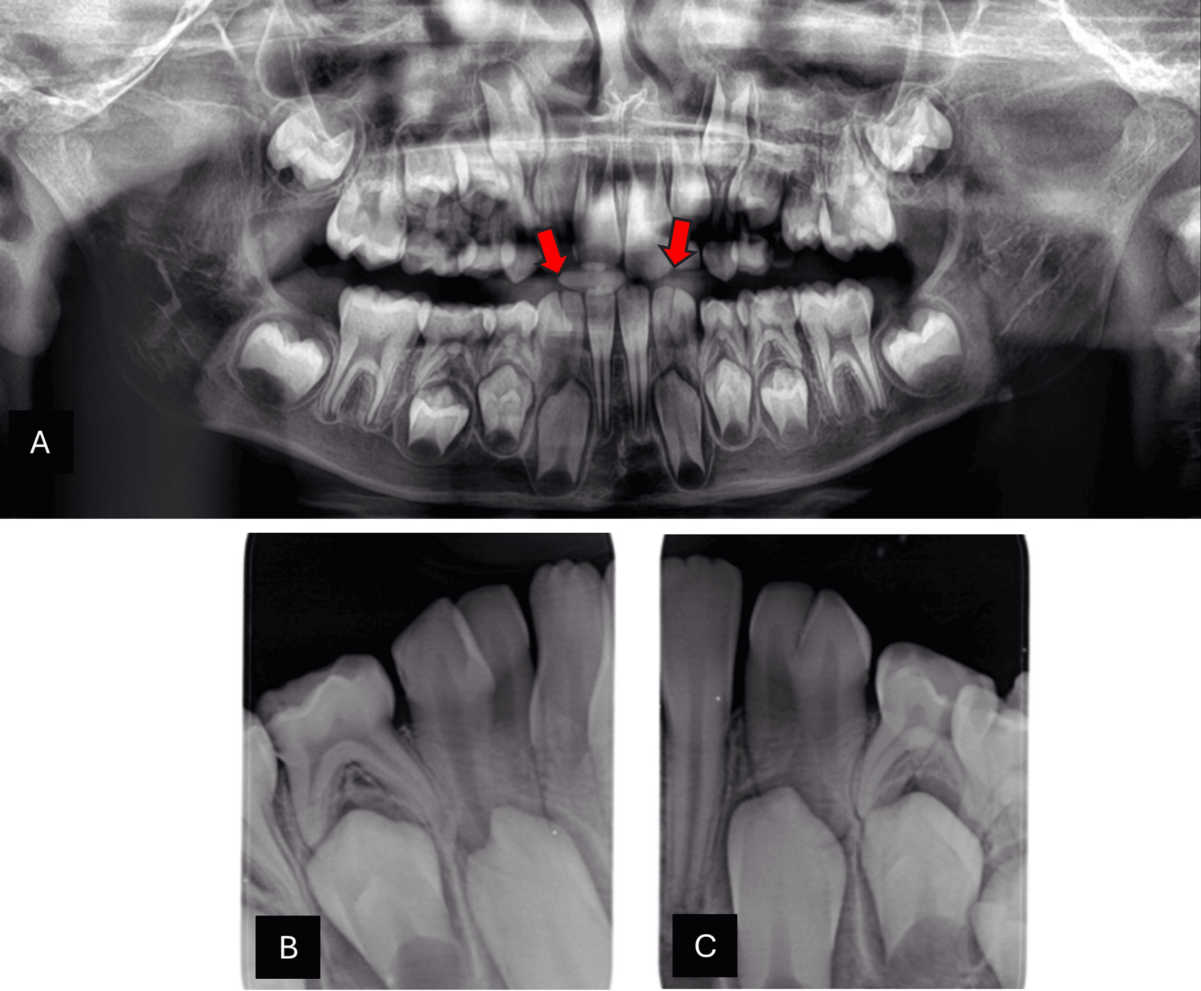
: Radiographic examination of case 2 (A) Panoramic radiograph: The red arrows highlight bilateral fusion at the mandibular arch in both right and left sides with congenitally missing successor lateral incisors. (B) Periapical radiograph: shows right side fusion of the teeth between primary lateral incisor and canine with congenitally missing successor lateral incisors. (C) Periapical radiograph: shows left side fusion of the teeth between primary lateral incisor and canine with congenitally missing successor lateral incisors.

Radiographic findings confirmed bilateral fusion and demonstrated congenitally missing permanent lateral incisors on both sides. The panoramic radiograph clearly highlights the bilateral fusion with red arrows marking the fused teeth (Figure 4A). The periapical views provide closer visualization of the right and left sides, confirming the union between the primary lateral incisors and canines and the absence of their permanent successors (Figures 4B, 4C).

Case three

**Figure 5:**
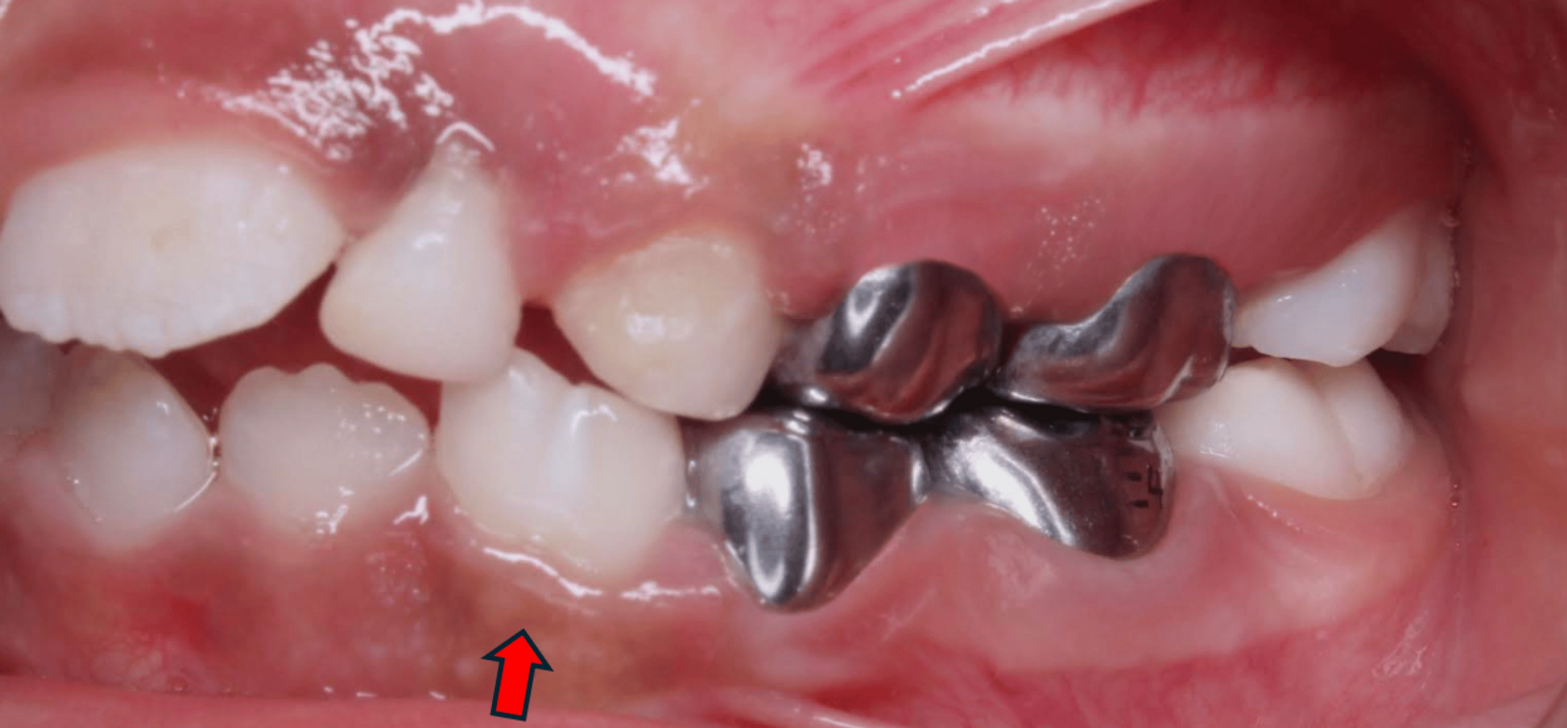
Intraoral view case 3 The red arrow point to the Unilateral fusion between mandibular primary lateral incisor and canine at the left side.

A seven-year-old girl attended the clinic for restorative care. She had no relevant medical history. Clinical examination showed poor oral hygiene, multiple carious teeth, and a history of previous dental treatment. A unilateral fusion was observed between the mandibular left primary lateral incisor and canine. The intraoral view demonstrates the fused crowns on the left side of the mandible, with the red arrow indicating the site of fusion (Figure 5).

**Figure 6:**
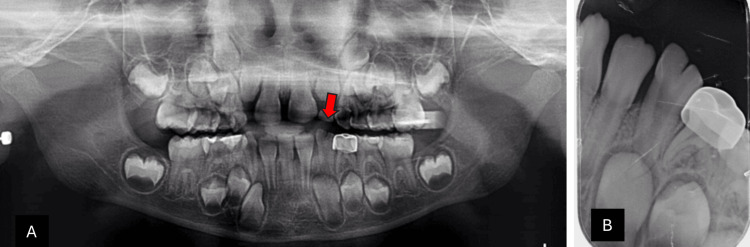
Radiographic examination of case 3 (A) Panoramic radiograph: the red arrow points to the unilateral fusion at the mandibular left side with congenitally missing successor lateral incisors. (B) Periapical radiograph: shows fusion between primary lateral incisor and canine with a congenitally missing successor lateral incisor.

Radiographic examination confirmed the fusion and showed hypodontia of the permanent mandibular left lateral incisor. The panoramic view demonstrates the unilateral fusion on the left side of the mandible, with the red arrow indicating the fused crowns (Figure 6A). The periapical radiograph provides a closer view of the union between the primary lateral incisor and canine, along with the absence of the permanent successor (Figure 6B).

Collectively, these cases illustrate a consistent presentation of fused mandibular primary lateral incisors and canines with congenitally missing permanent successors, underscoring the importance of early clinical recognition, radiographic follow-up, and timely management in affected children.

Treatment

In this phase, the management was primarily preventive, aiming to preserve function, esthetics, and arch space. Preventive measures included fluoride applications, fissure sealant placement, and long-term follow-up to ensure proper monitoring of dental development. The fused primary teeth were maintained until the eruption of the permanent successors, after which a definitive treatment plan, such as orthodontic intervention, space maintenance, or restorative options, would be tailored according to the specific clinical needs of the patient.

## Discussion

This report presents three rare cases of fused primary teeth associated with hypodontia of the permanent successors. In all cases, the fusion occurred in the mandibular anterior region between the lateral incisor and canine, resulting in a reduced tooth count. No caries were observed along the fusion grooves, and one case demonstrated bilateral fusion, which is particularly uncommon. Although the sample size is small, these findings are consistent with previous reports on this anomaly.

‏Differentiating between gemination, fusion, or a double tooth is not merely a matter of terminology but has important clinical implications. The overlapping features of these anomalies can lead to diagnostic uncertainty, which in turn affects treatment planning, prognosis, and communication between clinicians. For instance, while gemination typically preserves the normal tooth count, fusion often results in a reduced number of teeth, potentially creating arch asymmetry and space management challenges. Therefore, accurate differentiation, supported by radiographic evaluation, is critical to guide appropriate management and long-term follow-up [1-3].

The clinical features of fused teeth vary depending on whether the fusion is unilateral or bilateral and the extent of the union. Clinically, fused teeth often appear larger than normal and can be mistaken for gemination or macrodontia. Bilateral fusion, as observed in one of our cases, is exceptionally rare and has been linked to more complex occlusal and space management challenges than unilateral cases, emphasizing the need for close monitoring and individualized treatment planning [11,12].

Diagnosis is best established by combining clinical and radiographic evaluation. Clinically, fused teeth present as abnormally large crowns, sometimes with a developmental groove separating the two segments. Radiographs confirm the diagnosis and determine whether the pulp chamber and canals are shared or separate, helping to differentiate fusion from gemination. Accurate diagnosis is essential as it guides management and long-term monitoring of the developing dentition [6,8,13].

The etiology of fusion remains uncertain, although physical pressure between adjacent tooth buds during morphodifferentiation is the most accepted theory. Genetic and environmental factors have also been suggested, which may explain familial cases and associations with other anomalies. However, the condition is considered multifactorial, with no single cause definitively proven [2,6,8].

Management of fused primary teeth is generally conservative. Preventive strategies, including fluoride application, sealants, and regular follow-up, are crucial. When permanent successors are absent, retaining fused teeth helps preserve space until definitive treatment can be planned. Orthodontic or prosthetic options, such as implants or bridges, may later be considered as part of a multidisciplinary approach tailored to the child’s growth and clinical needs [13,14]. However, intervention becomes necessary if complications such as pulp necrosis, periapical infection, or functional disturbances arise. In such cases, extraction with space maintenance, orthodontic alignment, or prosthetic rehabilitation may be indicated. Definitive treatments, such as implants or fixed prostheses, are best postponed until craniofacial growth is complete to ensure long-term stability and esthetics. Thus, the timing of intervention is critical: delayed treatment risks space loss, midline shift, or esthetic issues, while premature extraction may compromise occlusion and function [8-14].

## Conclusions

Fused primary teeth are usually detected during routine examinations, and early recognition is essential for assessing their impact on dental development and permanent successors. The three cases in this series consistently involved mandibular primary lateral incisors and canines with congenitally missing successors, highlighting the importance of vigilant diagnosis and follow-up. A preventive approach was adopted to preserve function, esthetics, and arch space, with definitive treatment such as orthodontic or restorative interventions planned according to individual needs. Timely recognition and tailored management remain crucial for achieving stable functional and esthetic outcomes.
